# Association of Dual-Task Gait Cost and White Matter Hyperintensity Burden Poststroke: Results From the ONDRI

**DOI:** 10.1177/15459683231177606

**Published:** 2023-06-02

**Authors:** Frederico Pieruccini-Faria, Benjamin Cornish, Malcolm Binns, Julia Fraser, Seyyed M. H. Haddad, Kelly Sunderland, Joel Ramirez, Derek Beaton, Donna Kwan, Allison A. Dilliott, Christopher Scott, Yanina Sarquis-Adamson, Alanna Black, Karen Van Ooteghem, Leanne Casaubon, Dar Dowlatshahi, Ayman Hassan, Jennifer Mandzia, Demetrios Sahlas, Gustavo Saposnik, Brian Tan, Robert Hegele, Dennis Bulman, Mahdi Ghani, John Robinson, Ekaterina Rogaeva, Sali Farhan, Sean Symons, Nuwan Nanayakkara, Stephen R. Arnott, Courtney Berezuk, Melissa Holmes, Sabrina Adamo, Miracle Ozzoude, Mojdeh Zamyadi, Wendy Lou, Sujeevini Sujanthan, Robert Bartha, Sandra E. Black, Richard H. Swartz, William McIlroy, Manuel Montero-Odasso

**Affiliations:** 1 Gait and Brain Lab, St. Joseph’s Hospital, Parkwood Institute, Lawson Health Research Institute, Department of Medicine, Schulich School of Medicine and Dentistry, Western University, London, ON, Canada; 2 Neuroscience, Mobility and Balance Lab (NiMBaL), Department of Kinesiology and Health Sciences, University of Waterloo, Waterloo, ON, Canada; 3 Rotman Research Institute, Baycrest Centre, Toronto, ON, Cananda; Dalla Lana School of Public Health, University of Toronto, Toronto, ON, Canada; 4 Department of Medicine, Robarts Research Institute, Schulich of Medicine and Dentistry, Western University, London, Ontario, Canada; 5 Hurvitz Brain Sciences Program, Sunnybrook Research Institute, Toronto, ON, Canada; 6 Data Science & Advanced Analytics, St. Michael’s Hospital, Unity Health Toronto, Toronto, ON, Canada; 7 Centre for Neuroscience Studies, Queen’s University, Kingston, ON, Canada; 8 Department of Biochemistry, Schulich School of Medicine and Dentistry, Western University, London, ON, Canada; Robarts Research Institute, Western University, London, ON, Canada; 9 Department of Medicine, Sunnybrook HSC, University of Toronto; Dr. Sandra Black Centre for Brain Resilience and Recovery; Hurvitz Brain Sciences Research Program Director, Sunnybrook Research Institute; Heart and Stroke Foundation Canadian Partnership for Stroke Recovery Sunnybrook Health Sciences Centre, Toronto, ON, Canada; 10 Department of Medicine, University of Ottawa Brain and Mind Research Institute and Ottawa Hospital Research Institute, Ottawa, ON, Canada; 11 Thunder Bay Regional Research Institute, Northern Ontario School of Medicine, Thunder Bay, ON, Canada; 12 Department of Clinical Neurological Sciences, Schulich School of Medicine and Dentistry, Western University, and London Health Sciences Center, London, ON, Canada; 13 Division of Neurology, Department of Medicine, McMaster University, Hamilton, ON, Canada; 14 St. Michaels Hospital, University of Toronto, Toronto, ON, Canada; 15 Tanz Centre for Research in Neurodegenerative Diseases, University of Toronto, Toronto, ON, Canada; 16 Department of Neurology and Neurosurgery, Department of Human Genetics, The Montreal Neurological Institute, McGill University, Montreal, QC, Canada; 17 Dalla Lana School of Public Health; University of Toronto, Toronto, ON, Canada; 18 Department of Ophthalmology and Visual Sciences, Research Institute of the McGill University Health Center, Montreal, QC, Canada; 19 Department of Medicine (Geriatrics) and Epidemiology and Biostatistics, Schulich School of Medicine and Dentistry, Western University, London, ON, Canada

**Keywords:** gait, stroke, dual-task, white matter hyperintensities, atrophy, brain structure

## Abstract

**Background:**

Acute change in gait speed while performing a mental task [dual-task gait cost (DTC)], and hyperintensity magnetic resonance imaging signals in white matter are both important disability predictors in older individuals with history of stroke (poststroke). It is still unclear, however, whether DTC is associated with overall hyperintensity volume from specific major brain regions in poststroke.

**Methods:**

This is a cohort study with a total of 123 older (69 ± 7 years of age) participants with history of stroke were included from the Ontario Neurodegenerative Disease Research Initiative. Participants were clinically assessed and had gait performance assessed under single- and dual-task conditions. Structural neuroimaging data were analyzed to measure both, white matter hyperintensity (WMH) and normal appearing volumes. Percentage of WMH volume in frontal, parietal, occipital, and temporal lobes as well as subcortical hyperintensities in basal ganglia + thalamus were the main outcomes. Multivariate models investigated associations between DTC and hyperintensity volumes, adjusted for age, sex, years of education, global cognition, vascular risk factors, APOE4 genotype, residual sensorimotor symptoms from previous stroke and brain volume.

**Results:**

There was a significant positive global linear association between DTC and hyperintensity burden (adjusted Wilks’ λ = .87, *P* = .01). Amongst all WMH volumes, hyperintensity burden from basal ganglia + thalamus provided the most significant contribution to the global association (adjusted β = .008, η^2^ = .03; *P* = .04), independently of brain atrophy.

**Conclusions:**

In poststroke, increased DTC may be an indicator of larger white matter damages, specifically in subcortical regions, which can potentially affect the overall cognitive processing and decrease gait automaticity by increasing the cortical control over patients’ locomotion.

## Introduction

Mobility and cognitive impairments co-exist and have intertwined relationships in patients who experience vascular white matter lesions caused by a stroke.^1^ Current theoretical framework suggests that mobility and cognition share common brain networks that may be damaged by cerebrovascular disease.^2^ Dual-task gait cost (DTC), or the acute change in gait performance when simultaneously performing a mental task,^3,4^ was found to be higher in individuals with cognitive and motor syndromes. Furthermore, DTC is a predictor of motor^5,6^ and cognitive^7,8^ disabilities in individuals with history of stroke (poststroke) and accelerated brain degeneration.^10-12^ However, there is insufficient evidence of an independent association between DTC and hyperintensity signals (white matter lesions),^13^ particularly regarding its location, in Poststroke.

Increased DTC has been related to reduced cognitive^5,6,14^ and neural efficiency^15^ in frontal lobe regions in poststroke. A recent study, in healthy older adults, suggested that global microstructural white matter defects mediates the relationship between neural efficiency in prefrontal cortex and dual-task gait performance.^16^ White matter lesions are expected to reduce processing speed^17^ which consequently may affect the ability to walk and talk due to increased processing demands for task switching, as suggested by previous studies in Poststroke.^6,14^ White matter hyperintensity (WMH) is a typical macrostructural brain tissue alteration, detected with magnetic resonance imaging, in poststroke,^11,12,18-22^ and a strong predictor of fast progression to dementias,^18-21^ particularly when concentrated in the frontal lobe.^11,12,22^ Previous studies in older adults also have found global association between slow gait performance under dual-task conditions and larger WMH volumes and mostly focused on cortical regions.^23,24^ Notably, the investigation on the association between WMH burden in the parietal, occipital, temporal lobes and gait performance have been neglected, although pathology in these cortical regions can be directly or indirectly linked with mobility impairments.^25^ Moreover, despite subcortical regions, including basal ganglia and thalamus be known for its strong involvement with subconscious/well-learnt/automatic sensorimotor processing,^26-28^ studies also have shown that these subcortical regions are important to support cognitive processes occurring in frontal lobe regions during complex goal-directed actions.^26,27,29^ Hence, an independent association between subcortical WMH burden and increased DTC would suggest increased cognitive load for gait control driven by subcortical dysfunction (ie, decreased motor automaticity). However, it is unknown whether and how WMH burden in these regions would be associated with gait and DTC.

We hypothesize that increased DTC (ie, percentage of gait slowing from single- to dual-task testing conditions), will be cross-sectionally associated with larger WMH volumes in cortical and subcortical regions while controlling associations for potential confounders including atrophy.^30^ It is expected that increased DTC will be globally associated with larger WMH burden with greater contributions from aforementioned brain regions closely involved with complex thinking and sensorimotor processing for gait control. Importantly, because brain atrophy is quite prevalent in poststroke^31^ and strongly linked with worse gait performance in individuals with cerebrovascular disease,^30^ we also tested whether the normal appearing volumes would mediate the expected association between DTC and WMH. To test these hypotheses, we examined the cerebrovascular disease (CVD) cohort from the Ontario Neurodegenerative Disease Research Initiative (ONDRI),^32^ which is exclusively composed by patients with clinically confirmed history of stroke.

## Methods

### General Inclusion and Exclusion Criteria in ONDRI

Participants met each of the following criteria for enrolment into the ONDRI study: written informed consent obtained and documented; proficiency in speaking and understanding English at 7 out of 10 or higher on the 2 LEAP-Q questions; ≥ 8 years education; minimum Montreal Cognitive Assessment (MoCA)^33^ score of ≥18; ability to walk unassisted for at least 10 m; participant must have a reliable study partner.^34^

Participants were excluded if they: had serious underlying disease other than the disease being studied which in the opinion of the investigator interfered with the participant’s ability to participate fully in the study; had any disease that would/could lead to death over the next 3 to 5 years (ie, cardiac/renal/liver cancer) with poor prognosis; had a history of alcohol or drug abuse, which in the opinion of the investigator, interfered with the participant’s ability to comply with the study procedures; substance abuse within the past year; unstable cardiac, pulmonary, renal, hepatic, endocrine, hematologic, or active malignancy or infectious disease; AIDS or AIDS-related complex; unstable psychiatric illness defined as psychosis (hallucinations or delusions) or untreated major depression within 90 days of the screening visit; currently enrolled in a disease modifying therapeutic (drug or interventional) trial or observational study that the ONDRI Executive Committee felt would compromise study results; clinical diagnosis of glaucoma defined as taking eye-drops for glaucoma, or had surgery for glaucoma in 1 or both eyes or any other known serious eye disease (eg, wet/exudative age-related macular degeneration) or treatment or eye surgery including any history of intra-vitreal injections. Depending on the eye disease, the participant was excluded if the condition was present in 1 or both eyes. Participant has a known diagnosis of multiple sclerosis; history of optic neuritis or other optic neuropathy in 1 or both eyes; poorly controlled diabetes, defined by an A1c of 7.5% or higher obtained from the screening blood-work drawn; retinal laser therapy (either pan-retinal, or grid/focal) for diabetic retinopathy in 1 or both eyes.

### Clinical Evaluation of Stroke Etiology and Function

Stroke etiology was performed by experienced clinicians using the Etiology of stroke classified with TOAST (Trial of Org 10172 in Acute Stroke Treatment).^35^ The National Institute of Health Stroke Scale (NIHSS) was used to diagnose motor and sensory symptoms of a stroke in this cohort.

### Participants Excluded From the Final Data Analysis

Participants from the CVD cohort in ONDRI were excluded from the final analysis if presenting negative DTC (ie, walking faster while dual-tasking compared to usual gait) to avoid inclusion of potentially not dual-task compliant participants. Additionally, participants with missing data for gait, clinical data used as covariates, or missing neuroimaging metrics, were also excluded. This criterion helped us decrease overall data heterogeneity.

### ONDRI CVD Cohort Criteria

Participants in this cohort (N = 161) were within the range of 55 to 85 years of age.

#### Inclusion

Participants with an ischemic stroke event documented on MRI or CT and meet the following criteria:

(i) ≥ 3 months since stroke; (ii) mild-moderate stroke [modified Rankin Scale (mRS)^36^ must be 0-3]; (iii) without history of baseline dementia before stroke (pre-stroke mRS ≤2); and (iv) previous silent stroke (seen on CT or MRI but without clinical history of focal neurological deficits) allowed.

#### Exclusion

(i) Participant with no vascular cause of symptoms (eg, migraine and isolated vertigo); (ii) participant with large cortical strokes (>1/3 middle cerebral artery); (iii) participant with severe cognitive impairment, aphasia, inability to write, and/or severe functional disability limiting ability to perform assessments.

All participants included in this study read and signed written consent forms to participate. This study was approved by local research ethics board at University of Western Ontario, London-Canada (REB#104915).

#### Gait Protocol and Measurements

Gait performance was recorded with accelerometers attached bilaterally to participants’ ankles.^37^ Participants were invited to walk straight ahead, unassisted, for 8 meters at their usual pace under different conditions; however only the central 6 meters of the walking trial were used for analysis to remove positive and negative acceleration phases (first and last meter) and declaration (last meter) phases from gait performance.^4,37^ The average of 3 self-selected gait speed trials, and the gait speed during “naming animals” condition were examined. In this dual-task condition participants are instructed to say as many different animals as they can out loud while walking at their usual pace.^9^ The DTC was calculated as follows:

$$DTC=\frac{UGavg-DTAn}{UGavg}\times 100$$

Where *UGavg* is the average gait speed calculated from 3 trials and *DTAn* is the dual-task gait speed while naming animals.

### Neuroimaging Assessments and Metrics

All MRI data acquired as part of the ONDRI study were obtained on 3T scanners as described previously.^38^ using a protocol consistent with the Canadian Dementia Imaging Protocol The procedures used in generating tissues and regions of interest have been detailed in previous papers (Semi-Automatic Brain Region Extraction,^39^ Lesion Explorer,^40,41^ and FLEX^42^), including a scan-rescan reliability analysis.^43^ Briefly, interleaved Proton density (PD) and T2-weighted images (T2) and Fluid-attenuated inversion recovery (FLAIR) images were co-registered to the T1-weighted image and a PD-T2 based intra-cranial vault mask was automatically generated and manually edited to include supra-tentorial cerebrospinal fluid (CSF). Using this mask, the T1-weighted image was segmented using a multi-feature histogram method^44,45^ to segment tissue into grey matter, white matter, and CSF volumes (not presented in this study), at which point ventricular CSF (vCSF) was manually relabeled and the cerebellum was manually removed from the image. White matter hyperintensities from brain lobes, hyperintensities from basal ganglia and thalamus, and lacunes were automatically identified using FLEX (FLAIR-based) and Lesion Explorer (PD-T2-based) respectively and manual edits were applied to remove false positives and recover false negatives. Each lesion type was further subdivided by an automated algorithm into a region-based class (periventricular or deep) based on their 3-dimensional location by an automated algorithm.^40-42^ Further, Lesion Explorer was used to capture enlarged perivascular spaces (PVS),^32,37^ and cortical strokes were manually traced by trained neuroimaging analysts and supervised by a research neuroradiologist. A combination of these tissue segmentation methods produced 10 different tissue classes which consisted of: normal-appearing white matter, normal-appearing grey matter, sulcal CSF, vCSF, periventricular WMH, deep WMH, periventricular lacunes, deep lacunes, PVS, and strokes. For the purpose of this study, all volumes were summed up to calculate the total volumes of frontal, parietal, occipital and temporal lobes; and subcortical structures basal ganglia + thalamus. The percentage of WMH volume per lobe and in basal ganglia + thalamus was then calculated.

### Genomics

The Apolipoprotein E e4 genotype (ie, APOE4) was classified using a standardized procedure already described in the ONDRI protocol published elsewhere.^34^ Participants with at least 1 E4 allele are classified as APOE4 *carriers* whereas individuals without the E4 allele are classified as APOE4 *noncarriers* for analysis purposes.

### Covariates

Covariates included in our statistical analysis were defined *a priori* consistent with previous published studies^46^ as an attempt to decrease the biological, physiological, neurological and behavioral heterogeneity in the CVD cohort. Therefore, the covariates selected were: age, sex, years of education, global cognition (MoCA score), vascular risk factors (sum),^47^ APOE4 genotype, and residual sensorimotor symptoms from previous stroke identified with the NIHSS and total brain volume.

### Data Analysis

**Figure 1.**
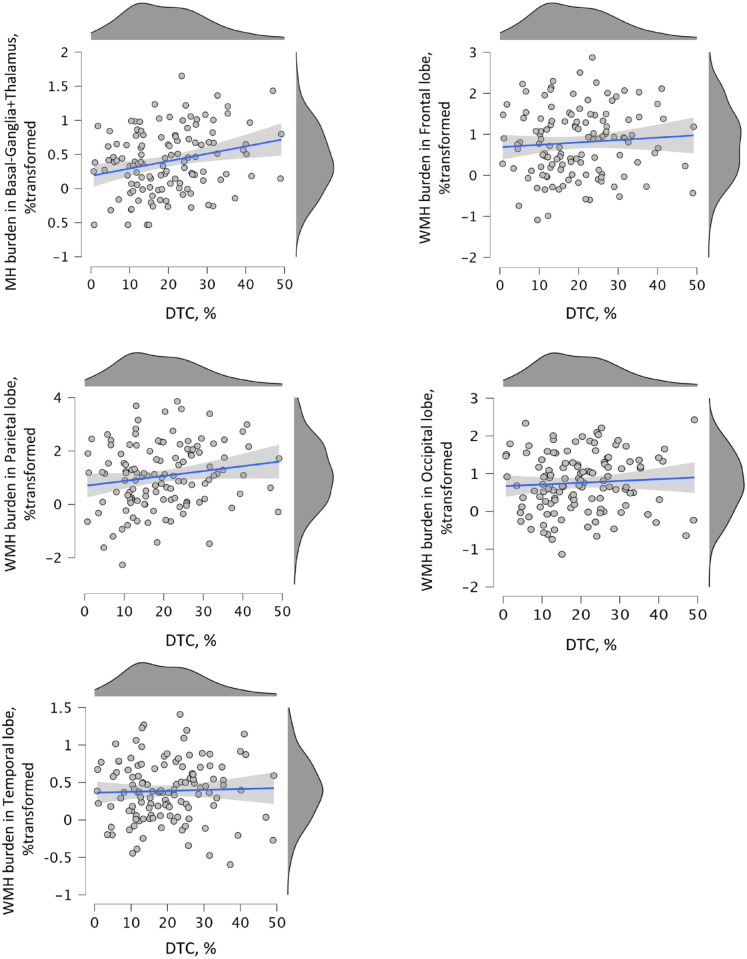
Scatter plots displaying linear associations between DTC and WMH burden in individuals with history of stroke (poststroke). Significant univariate association between basal ganglia + thalamus WMH volume (% transformed for normality) and dual task gait cost (DTC, %) as identified by the multivariate model.

Clinical, cognitive, gait and neuroimaging data were submitted to a rigorous quality control procedure^48^ using customized algorithms for automated detection of errors and outliers before entering statistical analysis. This dataset is described in tables through means, standard deviation and percentages. WMH volumes percentage in each region of interest (ie, burden) were used to better estimate the extension of white matter damage in each brain region for fairer comparisons across participants. Since WMH burden (outcome measure) in all regions were not normally distributed, they were transformed to achieve statistical normality using a 2-steps transformation procedure described elsewhere.^49^ Subsequently, Kolmogorov–Smirnov tests and histograms revealed statistical normality for transformed hyperintensity percentages (Figure 1). To test our hypothesis that DTC is associated with WMH burden, we created 2 multivariate models: (1) unadjusted (crude or without adjustments for covariates); (2) 1 fully adjusted for covariates (see Table 2). This approach allowed us to show to what extent that potential confounders (ie, covariates) affected associations. Global linear association between DTC and WMH burden are indicated by Wilks’ lambda values in Table 2. If global association between DTC and neuroimaging outcomes were significant (Wilks’ λ *P* < .05) then the parameter estimates were used to reveal brain regions that mostly contributed to the global linear association. To examine whether and how much atrophy affected the associations described above a mediation analysis was performed. The SPSS software v. 27 (IBM Inc., Chicago, USA) was used for variable transformations and inferential statistics. A mediation analysis was also performed using PROCESS plug-in for SPSS.^50^ Correlation figures were created in JASP (https://jasp-stats.org/)

## Results

### Participants’ Characteristics

Demographics and clinical characteristics are shown in Table 1 as means, standard deviations and percentages. During the exclusion process we detected 18 participants who walked faster during the dual-task condition, 18 individuals with missing data and 2 outliers were excluded from the sample for the final analysis (N = 123). Sub-analysis (Supplemental Table 5) revealed that, compared with individuals with positive DTC, individuals with negative DTC were on average significantly younger (66.9 years × 69 years of age; *P* = .05), walked significantly faster during the dual-task condition (0.99 m/s × 0.76 m/s; *P* < .001); and named 1 animal less compared with individuals with positive DTC (median: 6 × 7 words; *P* = .08); and had better global cognition (MoCA: 26.3 × 25.4; *P* = .003). Poststroke participants showed, on average, very low or no signs of motor, and/or sensory sequelae from a stroke event (less than the minimal scalar graduation of the NIHSS). Moreover, the great majority of participants in the poststroke group showed NIHHS equal 0 (67.5%) and no one had a score greater than 6. We found that 41.5% of individuals in the poststroke group had detectable stroke volumes assessed by CT scans. Finally, Supplemental Tables 1, 2, and 3 show the overall brain morphology expressed by the percentage of normal appearing grey and white matter burdens, respectively. Statistically transformed hyperintensity burden distributions (for normality) per brain region assessed in the current study is shown in Figure 1. Statistical normality was obtained after data transformation therefore parametric statistics were applied to test our hypotheses.

**Table 1.** Characteristics of Older Individuals With History of Stroke (Poststroke).

Groups characteristics	Poststroke^#^ (n = 123)
Age in years, mean ± SD; min–max	69.0 ± 7.4; 54–85
Women, n (%)	36 (29)
Years of education, mean ± SD; min–max	14.8 ± 2.9; 8–20
MoCA (0-30), mean ± SD; min–max	25.4 ± 2.9; 18–30
VRI (0-5), mean ± SD; min–max	2.5 ± 1.1; 0–5
Hypertension, n (%)	91 (74)
Diabetes, n (%)	27 (22)
High cholesterol, n (%)	97 (51.2)
Obesity, n (%)	34 (27.6)
Smoking history, n (%)	63 (51.2)
APOE4 genotype, n (%)	29 (23.6)
NIHSS (0-24), mean ± SD; min–max	0.65 ± 0.93; 0–6
Usual gait speed m/s, mean ± SD; min–max	0.95 ± 0.25; 0.42–1.6
Dual-task gait speed (naming animals) m‎/s, mean ± SD; min–max	0.76 ± 0.22; 0.30–1.4
Dual task gait cost %, mean ± SD; min–max	19.8 ± 10.6; 0.70–49
Total animals named while walking, median; min–max	7; 3–12

1-way ANOVA or chi-square.

Abbreviations: MoCA, Montreal Cognitive Assessment; VRI, vascular risk index is calculated as the sum of risk factors for stroke including hypertension, diabetes, high cholesterol, obesity and smoking history; APOE4, Apolipoprotein E e4 carrier; NIHSS, National Institute of Health Stroke Scale; NA, not applicable.

# Cerebrovascular disease cohort in ONDRI.

### Association Between DTC and WMH Burden Poststroke

Multivariate linear regression models were used to test whether there was a positive association between increased DTC and larger hyperintensity burden (ie, percentages per region) in poststroke (Table 2). A significant global linear association between DTC and hyperintensity burden was found in both crude (Wilks’ λ = .85, *P* = .002) and fully adjusted (Wilks’ λ = .87; *P* = .01) models. These models revealed that DTC and hyperintensity burden across brain regions share 15% of their variance, decreasing to 13% when covariates were added to the model. Therefore, the set of covariates only explained 2% of the global variance in the unadjusted model. Unadjusted parameters estimate of univariate analysis revealed that hyperintensity burden was positively associated with all burdens, but only significantly with basal ganglia + thalamus (unadjusted β = .011, 95% confidence interval [CI] 0.003 to 0.018; η^2^ = .061; *P* = .006/adjusted β = .008, 95% CI 0.0001 to 0.016, η^2^ = .035, *P* = .04). Figure 1 shows the positive linear relationship between DTC and hyperintensity burden in basal ganglia + thalamus.

**Table 2.** Association Between DTC and WMH Volumes (Normalized Percentages) in Crude and Fully Adjusted Multivariate Models.

Multivariate regression analysis poststroke (n = 123)	DTC Wilks’ Lambda = **85, *P*** **=** .**002** unadjusted model		DTC Wilks’ λ = .**87, *P*** **=** .**01** model fully adjusted^ψ^	
Hyperintensity percentage per lobe and subcortical regions (outcome)	Parameter estimates β, 95%CI, η^2^	*P*-value	Parameter estimates β, 95%CI, η^2^	*P*-value
WMH volume in frontal lobe, %	.006, (−.008 to .019), .006	.40	.001, (−.013 to .012), .0001	.93
WMH volume in parietal lobe, %	.018, (−.002 to .038), .027	.07	.010, (−.007 to .029), .012	.25
WMH volume in occipital lobe, %	.004, (−.009 to .017), .003	.53	.002, (−.011 to .014), .0001	.79
WMH volume in temporal lobe, %	.001, (−.006 to .008), .001	.78	−.001, (−.008 to .005), .002	.66
WMH volume in basal ganglia + thalamus, %	.011, (.003 to .018), .061	.**006**	.008, (.0001 to .016), .035	.**04**

Bold values indicate statistically significant associations at *P* < .05.

ψ Multivariate model adjusted for age, sex, years of education, APOE4 genotype, NIHS score, MoCA, Vascular risk index, and intracranial volume.

### Association Between Single- and Dual-Task Gait Speed and WMH Burden Poststroke

Two additional multivariate models were performed to determine whether single- and dual-task gait speed would be associated with WMH burden. However, both models did not show significant global association with WMH burden (Single-task gait speed–adjusted crude Wilks’ λ = .95, *P* = .35; Wilks’ λ = .97, *P* = .70/Dual-task gait speed–crude Wilks’ λ = .95, *P* = .36; adjusted Wilks’ λ = .98, *P* = .88).

### Does Brain Atrophy Mediate Associations Between DTC and WMH Burden?

The mediation analysis did not reveal any significant indirect effects of brain tissue atrophy on the association between DTC and hyperintensity burden (supplemental Table 4). Therefore, associations described above are unaffected by changes in normal appearing white and gray matter volumes.

## Discussion

This observational cross-sectional cohort study showed in a large sample of poststroke participants that increased DTC may be a marker of greater WMH burden, particularly in subcortical regions (basal ganglia + thalamus). This association was independent of brain atrophy as revealed by our mediation analysis. Overall, our findings suggest that increased DTC is directly or indirectly linked with damage in cortical and subcortical networks.

Our findings showed significant associations between increased DTC and global WMH burden with greater significant contributions of subcortical structures when compared with brain lobes. These results suggest that cortical and subcortical white matter lesions may affect the ability to walk and talk not only because of cognitive impairment driven by cortical damage, but also due to a potential loss of gait automaticity. This is because subcortical regions are crucial for well-learnt sensorimotor processes including gait.^51,52^ Hence, increased DTC may express the increased cognitive demand of gait probed with DTC.^51,53^ Notably, in our study, the association between WMH was only significant with DTC, but not with gait speed. This also suggests that white matter lesions are strictly or mostly related with the cognitive processing of a dual-task while walking. Therefore, besides the known link between cognitive impairment and poorer dual-task performance after stroke events,^6,7^ our results suggest a stronger link between impaired cognitive processing, walking automaticity and vascular lesions in white matter, especially when concentrated in subcortical regions. To our knowledge this is the first study describing this potential mechanism in Poststroke.

Interpretations regarding associations between a potential loss of automaticity and WMH coming from our study should be taken cautiously because previous studies have found associations between gait performance, white matter lesions^17^ and poorer executive functions.^5,6,14^ It is still unclear, therefore, to which degree cognition and automaticity would affect cortical control of gait in this population, although it is expected that both would negatively affect gait performance.^52,54^ From a mechanistic perspective, it would be important that future studies be designed to investigate whether and how cognitive domains (eg, attention, executive function, memory, visuospatial, and language) mediate/explain the associations between DTC and WMH, so that more accurate cause-effects inferences can be made.

Another important result is that atrophy did not affect the association between DTC and WMH burden. This result may be in disagreement with Briley et al^30^ that showed that brain atrophy attenuates the association between gait dysfunction and white matter lesions in older adults. It is important to mention, however, that in the study by Briley et al the authors evaluated usual gait performance using a clinical scale in a much more clinically heterogenous sample (not necessarily poststroke). Therefore, our results would be better applied to individuals with history of stroke (NIHSS 0-6), rather than older adults in general.

The current study has several strengths including a reliable and comprehensive protocol of assessments. This study also controlled the analysis for potential physical, cognitive and biological confounders that could affect both DTC and white matter integrity that were not addressed in previous studies. We acknowledge, however, that some white matter tracts and brain sub regions, not assessed in the current study, including the pre-motor cortex, motor cortex, putamen, caudate and thalamic radiations may be more important for motor-cognitive interactions than other regions, but because of methodological limitations in the ONDRI neuroimaging pipeline it was not possible to include them in this study. Therefore, this regional specificity should be scrutinized in future studies using larger samples. Due to few detected strokes in current study, it was not possible to assess the impact of stroke location on gait although we acknowledge that it may contribute to more specific and severe sensorimotor impairments. The lack of an age-matched control group makes it difficult to determine whether WMH volumes found in our poststroke participants are different from healthy individuals’ volumes without history of stroke in general. Finally, the cross-sectional analysis in the current study limits conclusions regarding cause-effect relationships and predictions.

## Conclusion

Increased DTC is associated with larger global WMH burden in individuals with history of stroke. WMH when located in subcortical regions, specifically basal ganglia and thalamus, may have greater contributions to multitasking difficulties while walking. This would also suggest a potential mechanism of loss of gait automaticity in Poststroke which may be expressed as increased DTC.

## Supplemental Material

sj-docx-1-nnr-10.1177_15459683231177606 – Supplemental material for Association of Dual-Task Gait Cost and White Matter Hyperintensity Burden Poststroke: Results From the ONDRI Click here for additional data file. Supplemental material, sj-docx-1-nnr-10.1177_15459683231177606 for Association of Dual-Task Gait Cost and White Matter Hyperintensity Burden Poststroke: Results From the ONDRI by Frederico Pieruccini-Faria, Benjamin Cornish, Malcolm Binns, Julia Fraser, Seyyed M. H. Haddad, Kelly Sunderland, Joel Ramirez, Derek Beaton, Donna Kwan, Allison A. Dilliott, Christopher Scott, Yanina Sarquis-Adamson, Alanna Black, Karen Van Ooteghem, Leanne Casaubon, Dar Dowlatshahi, Ayman Hassan, Jennifer Mandzia, Demetrios Sahlas, Gustavo Saposnik, Brian Tan, Robert Hegele, Dennis Bulman, Mahdi Ghani, John Robinson, Ekaterina Rogaeva, Sali Farhan, Sean Symons, Nuwan Nanayakkara, Stephen R. Arnott, Courtney Berezuk, Melissa Holmes, Sabrina Adamo, Miracle Ozzoude, Mojdeh Zamyadi, Wendy Lou, Sujeevini Sujanthan, Robert Bartha, Sandra E. Black, Richard H. Swartz, William McIlroy and Manuel Montero-Odasso in Neurorehabilitation and Neural Repair
