# Supplementary material for: Association of Dual-Task Gait Cost and White Matter Hyperintensity Burden Poststroke: Results From the ONDRI
Source: Neurorehabil Neural Repair. 2023 Jun 2;37(7):434–43. doi: 10.1177/15459683231177606 (PMC10350733; doi:10.1177/15459683231177606)
Supplement: sj-docx-1-nnr-10.1177_15459683231177606 – Supplemental material for Association of Dual-Task Gait Cost and White Matter Hyperintensity Burden Poststroke: Results From the ONDRI [file sj-docx-1-nnr-10.1177_15459683231177606.docx]

**SUPPLEMENTARY MATERIAL**

Supplementary Table 1 – Regional brain volumes in each group.

|  | Poststroke(n=123) |
| --- | --- |
| Tissue volumes/Neuroimaging metrics | mean±SD |
| Total volume per region^*^: |  |
| Frontal lobe volume, cm^3^ | 429.6±607.7 |
| Parietal lobe volume, cm^3^ | 324.4±371.3 |
| Occipital lobe volume, cm^3^ | 119.2±151.8 |
| Temporal lobe volume, cm^3^ | 288.4±334.6 |
| Basal ganglia+thalamus volume, cm^3^ | 577.9±605.1 |
| Intracranial volume, cm^3^ | 1225.4±135.8 |
| Hyperintensity volumes: |  |
| WMH in frontal lobe, cm^3^ | 3.7±5.5 |
| WMH in parietal lobe, cm^3^ | 3.6±5.7 |
| WMH in occipital lobe, cm^3^ | 1.0±1.0 |
| WMH in temporal lobe, cm^3^ | 1.2±1.6 |
| WMH in basal ganglia+thalamus, cm^3^ | 0.2±0.3 |

*Note* – WMH , White matter hyperintensities; *, total volume calculated as the sum of normal appearing white matter, normal appearing grey matter, cerebrospinal fluid volume, lacunes volume, periventricular spaces volume and white matter hyperintensities volume. See methods section for detailed description of brain tissues segmentation.

Supplementary Table 2 –Hyperintensity percentages transformed for normality in brain lobes and subcortical structures.

|  | Poststroke  (n=123) |  |
| --- | --- | --- |
| Hyperintensity burden, % | mean±SD |  |
| WMH burden in frontal lobe, % | .78±.81 |  |
| WMH burden in parietal lobe, % | 1.03±1.20 |  |
| WMH burden in occipital lobe, % | .73±.75 |  |
| WMH burden in temporal lobe, % | .38±.39 |  |
| WMH burden in basal ganglia+thalamus, % | .39±.46 |  |

*Note* – ^ψ^, Group comparisons adjusted for age, sex, years of education, vascular risk factors, APOE4 genotype and intracranial volume; WMH, white matter hyperintensity.

Supplemental Table 3 – Percentage of normal appearing matter in major brain regions in individuals with history of stroke(Poststroke).

|  | Poststroke(n=123) |  |
| --- | --- | --- |
| NAM, % | mean±SD |  |
| NAM in frontal lobe, % | 71±5 |  |
| NAM in in parietal lobe, % | 74±5 |  |
| NAM in occipital lobe, % | 81±4 |  |
| NAM in temporal lobe, % | 80±5 |  |
| NAM in basal ganglia+thalamus, % | 75±6 |  |

*Note* – NAM, normal appearing matter(grey+white)

Supplemental Table 4 – Mediation effects of NAM percentage from each region on the association between DTC and basal ganglia+thalamus hyperintensity burden. No significant mediation effects were found.

| Mediation effect | Effect | Std.Error | Lower CI | Upper CI |
| --- | --- | --- | --- | --- |
| TOTAL | -0.0004 | 0.002 | -0.0037 | 0.0041 |
| NAM in frontal lobe**, %** | 0.0002 | 0.002 | -0.0038 | 0.0042 |
| NAM in in parietal lobe**, %** | 0.0002 | 0.0012 | -0.0016 | 0.0037 |
| NAM in occipital lobe**, %** | -0.0003 | 0.0008 | -0.0022 | 0.0011 |
| NAM in temporal lobe**, %** | -0.0011 | 0.0014 | -0.0039 | 0.002 |
| NAM in basal ganglia+thalamus**, %** | 0.0004 | 0.0011 | -0.0014 | 0.0033 |

Note – NAM, normal appearing matter(grey+white); CI, Confidence intervals

Supplementary Table 5 – Characteristics of older excluded individuals with *negative* DTC with history of stroke (Poststroke).

| Groups characteristics |  | Poststroke^#^ (n=18) |
| --- | --- | --- |
| Age in years, mean±SD; min~max |  | 66.9±7.5;52~83* |
| Women, n(%) |  | 5(27.8) |
| Years of education, mean±SD; min~max |  | 14.5±2.5;12~20 |
| MoCA (0-30), mean±SD; min~max |  | 26.3±2.5;22~30* |
| VRI (0-5), mean±SD; min~max |  | 2.6±1.1;0~5 |
| Hypertension, n(%) |  | 13(72.2) |
| Diabetes, n(%) |  | 2(11.1) |
| High cholesterol, n(%) |  | 13(72.2) |
| Obesity, n(%) |  | 7(38.9) |
| Smoking history, n(%) |  | 13(72.2) |
| APOE4 genotype, n(%) |  | 3(16.7) |
| NIHSS(0-24), mean±SD; min~max |  | 0.56±1.2;0~3 |
| Usual gait speed m/s, mean±SD; min~max |  | 0.91±0.24;0.59~1.6 |
| Dual-task gait speed(naming animals) m‎/s, mean±SD; min~max |  | 0.99±0.28;0.59~1.6* |
| Dual task gait cost %, mean±SD; min~max |  | -8.5±8.5; -24.3~ -.11* |
| Total animals named while walking, median; min~max |  | 6; 3~8 |

*Note* –One-way ANOVA or Chi-square; MoCA, Montreal Cognitive assessment; VRI, Vascular risk index is calculated as the sum of risk factors for stroke including hypertension, diabetes, high cholesterol, obesity and smoking history; APOE4, Apolipoprotein E e4 carrier; NIHSS, National Institute of Health Stroke Scale; NA, not applicable; ^#^, Cerebrovascular disease cohort in ONDRI.*, statistically different than positive DTC group.

Supplementary Table 6 – Association between *age* and WMH volumes (normalized percentages) in fully adjusted multivariate models.

| Multivariate Regression Analysis Poststroke(n=123) |  |  | DTC Wilks’ Lambda = .76, p=<.001  Model fully adjusted^ψ^ | |
| --- | --- | --- | --- | --- |
| Hyperintensity percentage per lobe and subcortical regions (outcome) |  |  | Parameter estimates  Beta, 95%CI, Eta^2^ | P-value |
| WMH volume in frontal lobe, % |  |  | 0.009,(0.03to0.067),0.201 | <.001 |
| WMH volume in parietal lobe, % |  |  | 0.013,(0.05to0.103)0.233 | <.001 |
| WMH volume in occipital lobe, % |  |  | 0.009,(0.015to0.052)0.101 | <.001 |
| WMH volume in temporal lobe, % |  |  | 0.005,(0.014to0.033)0.189 | <.001 |
| WMH volume in basal ganglia+thalamus, % |  |  | 0.006,(-0.001to0.022)0.028 | 0.075 |

Supplementary Table 7 – Association between *sex* and WMH volumes (normalized percentages) in fully adjusted multivariate models.

| Multivariate Regression Analysis Poststroke(n=123) |  |  | DTC Wilks’ Lambda = .87, p=.01  Model fully adjusted^ψ^ | |
| --- | --- | --- | --- | --- |
| Hyperintensity percentage per lobe and subcortical regions (outcome) |  |  | Parameter estimates  Beta, 95%CI, Eta^2^ | P-value |
| WMH volume in frontal lobe, % |  |  | 0.18,(0.234to0.947)0.088 | 0.001 |
| WMH volume in parietal lobe, % |  |  | 0.259,(0.265to1.293)0.075 | 0.003 |
| WMH volume in occipital lobe, % |  |  | 0.184,(-0.385to0.346)0 | 0.916 |
| WMH volume in temporal lobe, % |  |  | 0.091,(0.01to0.369)0.038 | 0.039 |
| WMH volume in basal ganglia+thalamus, % |  |  | 0.116,(0.043to0.502)0.048 | 0.02 |

Supplementary Table 8 – Association between *years of education* and WMH volumes (normalized percentages) in fully adjusted multivariate models.

| Multivariate Regression Analysis Poststroke(n=123) |  |  | DTC Wilks’ Lambda = .96, p=.52  Model fully adjusted^ψ^ | |
| --- | --- | --- | --- | --- |
| Hyperintensity percentage per lobe and subcortical regions (outcome) |  |  | Parameter estimates  Beta, 95%CI, Eta^2^ | P-value |
| WMH volume in frontal lobe, % |  |  | 0.023,(-0.065to0.024)0.007 | 0.371 |
| WMH volume in parietal lobe, % |  |  | 0.032,(-0.103to0.026)0.012 | 0.238 |
| WMH volume in occipital lobe, % |  |  | 0.023,(-0.091to0.001)0.033 | 0.053 |
| WMH volume in temporal lobe, % |  |  | 0.011,(-0.037to0.008)0.014 | 0.213 |
| WMH volume in basal ganglia+thalamus, % |  |  | 0.014,(-0.025to0.032)0.001 | 0.794 |

Supplementary Table 9 – Association between *MoCA* and WMH volumes (normalized percentages) in fully adjusted multivariate models.

| Multivariate Regression Analysis Poststroke(n=123) |  |  | DTC Wilks’ Lambda = .96, p=.59  Model fully adjusted^ψ^ | |
| --- | --- | --- | --- | --- |
| Hyperintensity percentage per lobe and subcortical regions (outcome) |  |  | Parameter estimates  Beta, 95%CI, Eta^2^ | P-value |
| WMH volume in frontal lobe, % |  |  | 0.024,(-0.028to0.066)0.006 | 0.426 |
| WMH volume in parietal lobe, % |  |  | 0.034,(-0.031to0.104)0.01 | 0.287 |
| WMH volume in occipital lobe, % |  |  | 0.024,(-0.064to0.032)0.004 | 0.506 |
| WMH volume in temporal lobe, % |  |  | 0.012,(-0.016to0.031)0.003 | 0.535 |
| WMH volume in basal ganglia+thalamus, % |  |  | 0.015,(-0.033to0.027)0 | 0.827 |

Supplementary Table 10 – Association between *NIHSS* and WMH volumes (normalized percentages) in fully adjusted multivariate models.

| Multivariate Regression Analysis Poststroke(n=123) |  |  | DTC Wilks’ Lambda = .92, p=.11  Model fully adjusted^ψ^ | |
| --- | --- | --- | --- | --- |
| Hyperintensity percentage per lobe and subcortical regions (outcome) |  |  | Parameter estimates  Beta, 95%CI, Eta^2^ | P-value |
| WMH volume in frontal lobe, % |  |  | 0.07,(0.007to0.283)0.038 | 0.039 |
| WMH volume in parietal lobe, % |  |  | 0.1,(0.054to0.452)0.054 | 0.013 |
| WMH volume in occipital lobe, % |  |  | 0.071,(-0.141to0.141)0 | 0.998 |
| WMH volume in temporal lobe, % |  |  | 0.035,(-0.021to0.118)0.017 | 0.17 |
| WMH volume in basal ganglia+thalamus, % |  |  | 0.045,(-0.07to0.107)0.002 | 0.677 |

Supplementary Table 11 – Association between *VRI* and WMH volumes (normalized percentages) in fully adjusted multivariate models.

| Multivariate Regression Analysis Poststroke(n=123) |  |  | DTC Wilks’ Lambda = .97, p=.74  Model fully adjusted^ψ^ | |
| --- | --- | --- | --- | --- |
| Hyperintensity percentage per lobe and subcortical regions (outcome) |  |  | Parameter estimates  Beta, 95%CI, Eta^2^ | P-value |
| WMH volume in frontal lobe, % |  |  | 0.059,(-0.105to0.127)0 | 0.853 |
| WMH volume in parietal lobe, % |  |  | 0.085,(-0.11to0.225)0.004 | 0.5 |
| WMH volume in occipital lobe, % |  |  | 0.06,(-0.114to0.125)0 | 0.93 |
| WMH volume in temporal lobe, % |  |  | 0.03,(-0.023to0.094)0.013 | 0.236 |
| WMH volume in basal ganglia+thalamus, % |  |  | 0.038,(-0.057to0.093)0.002 | 0.636 |

Supplementary Table 12 – Association between *APOE4* and WMH volumes (normalized percentages) in fully adjusted multivariate models.

| Multivariate Regression Analysis Poststroke(n=123) |  |  | DTC Wilks’ Lambda = .97, p=.68  Model fully adjusted^ψ^ | |
| --- | --- | --- | --- | --- |
| Hyperintensity percentage per lobe and subcortical regions (outcome) |  |  | Parameter estimates  Beta, 95%CI, Eta^2^ | P-value |
| WMH volume in frontal lobe, % |  |  | 0.153,(-0.522to0.084)0.018 | 0.154 |
| WMH volume in parietal lobe, % |  |  | 0.22,(-0.756to0.118)0.018 | 0.151 |
| WMH volume in occipital lobe, % |  |  | 0.157,(-0.513to0.108)0.015 | 0.199 |
| WMH volume in temporal lobe, % |  |  | 0.077,(-0.229to0.077)0.009 | 0.325 |
| WMH volume in basal ganglia+thalamus, % |  |  | 0.098,(-0.299to0.091)0.01 | 0.292 |

Supplementary Table 13 – Association between *intracranial volume* and WMH volumes (normalized percentages) in fully adjusted multivariate models.

| Multivariate Regression Analysis Poststroke(n=123) |  |  | DTC Wilks’ Lambda = .89, p=.03  Model fully adjusted^ψ^ | |
| --- | --- | --- | --- | --- |
| Hyperintensity percentage per lobe and subcortical regions (outcome) |  |  | Parameter estimates  Beta, 95%CI, Eta^2^ | P-value |
| WMH volume in frontal lobe, % |  |  | 0.0000005887,(0.0000007674to0.0000031)0.089 | 0.001 |
| WMH volume in parietal lobe, % |  |  | 0.000000849,(0.0000007089to0.000004074)0.067 | 0.006 |
| WMH volume in occipital lobe, % |  |  | 0.0000006037,(-0.0000005935to0.000001799)0.009 | 0.32 |
| WMH volume in temporal lobe, % |  |  | 0.0000002971,(0.0000001161to0.000001294)0.048 | 0.019 |
| WMH volume in basal ganglia+thalamus, % |  |  | 0.0000003787,(-0.0000005776to0.0000009234)0.002 | 0.649 |
